# Influence of Dexamethasone Premedication on Acute Lung Toxicity in Lung SBRT

**DOI:** 10.3389/fonc.2022.837577

**Published:** 2022-02-28

**Authors:** Fiori Alite, Parvez M. Shaikh, Anand Mahadevan

**Affiliations:** ^1^ Department of Radiation Oncology, Geisinger Cancer Institute, Danville, PA, United States; ^2^ Department of Radiation Oncology, West Virginia University, Morgantown, WV, United States

**Keywords:** stereotactic body radiation therapy (SBRT), lung, immunotherapy, dexamethasone, NSCLC

## Abstract

**Introduction:**

The cooperative group experience of thoracic sterotactic body radiation therapy (SBRT) in medically inoperable patients with early stage non-small cell lung cancer (NSCLC) historically utilized corticosteroid premedication. Patterns of care have been mixed as to whether premedication adds benefit in terms of improved lung toxicity and treatment tolerance.

**Methods:**

Patients treated for NSCLC from 2014 to 2017 with definitive thoracic SBRT (BED_10_≥100) at a single institution, in a prospectively collected database were evaluated. Pretreatment clinicopathologic characteristics, including Eastern Cooperative Oncology Group (ECOG) performance status, PFT parameters of FEV1, and diffusing capacity for carbon monoxide (DLCO) were collected. Treatment and dosimetric characteristics were collected, and patients were scored as to whether dexamethasone was prescribed and utilized with each fraction. Toxicity was graded on multiple domains including lung as during and 30 days after completion of treatment using Common Terminology Criteria for Adverse Events Version 4. Univariate analysis was performed with Fisher’s exact test for categorical variables and two-tailed Student’s *t*-test for continuous variables. Multivariate analysis was performed with Cox proportional hazards model to adjust for age, pretreatment DLCO, ECOG, tumor size, central versus peripheral location, and biological effective dose.

**Results:**

A total of 86 patients treated with thoracic SBRT with 54–60 Gy in 3–8 fractions met inclusion criteria, with the majority (70%) receiving 5 fractions. Of these patients, 45 (52%) received 4 mg dexamethasone premedication prior to each fraction of SBRT and 41 (48%) were treated without dexamethasone premedication. Overall acute lung toxicity was low in both groups. Between the two groups of patients, 5/45 (11%) developed grade 2 or higher lung toxicity including hospital admission in the dexamethasone premedication arm vs. 2/41 (5%) without premedication (*p* = 0.4370, Fisher’s exact test). Freedom from acute SBRT lung toxicity was no different between dexamethasone premedication arm and no premedication (Log rank, *p* = 0.45). On multivariate Cox proportional hazard modeling adjusting for age, ECOG, tumor size, central vs. peripheral location, pretreatment DLCO, and BED, there was no difference in freedom from acute lung toxicity without dexamethasone premedication (HR: 0.305; 95% CI: 0.033, 2.792; *p* = 0.293).

**Conclusions:**

In this retrospective analysis, pretreatment steroid prophylaxis with dexamethasone confers a similar acute toxicity profile and no added clinical benefit to treatment without pretreatment steroid prophylaxis.

## Introduction

Sterotactic body radiation therapy (SBRT) has evolved as the standard of care for medically inoperable early stage non-small cell lung cancer (NSCLC) and is being increasingly employed to ablate oligometastatic sites of disease progression to the lungs (1, 2). Early historical experiences with SBRT of 1–3 fractions generally employing higher doses of 18–30 Gy per fraction had consistently utilized corticosteroid premedication prior to each session of radiation (3–5). The use of oral and inhaled corticosteroids to prevent and/or treat acute and posttreatment radiation-induced lung injury is well established in clinical practice of conventionally fractionated thoracic radiation (6, 7). Early protocols of thoracic SBRT espoused that the expected acute inflammatory effects of large dose per fraction to lung parenchyma could be potentially abated by the use of steroid prophylaxis (4). Corticosteroid premedication, 15–60 min prior to each fraction of radiation, was mandated in the first phase II cooperative group trial (Radiation Therapy Oncology Group (RTOG) 0236) and has influenced the pattern of practice among treating radiation oncologists (5). Some rationale for this approach can be extrapolated from the available randomized evidence showing the benefit of corticosteroid prophylaxis administration in mitigating the acute inflammatory effects of radiation and thus preventing “pain-flare” phenomenon when utilizing single-fraction stereotactic radiation therapy for metastatic spinal metastases (8–10). However, whether the use of corticosteroid prophylaxis results in clinically meaningful improvements in the prevention of acute lung injury and therefore clinical toxicity has not been analyzed or reported on before in the literature. Although prophylactic treatment with corticosteroids may be a safeguard to prevent acute lung injury, mechanistic immune interactions of corticosterioid use may implicate efficacy of the programmed cell death 1 inhibitor (PD-1) inhibitors, which are being increasingly integrated with stereotactic ablative forms of radiation in all stages of NSCLC. We aimed to evaluate whether corticosteroid prophylaxis influenced the development of acute lung toxicity in patients receiving thoracic SBRT in our multicenter research database.

## Materials and Methods

Our multicenter prospectively collected institutional database of lung SBRT approved by the institutional review and privacy boards (IRB Number G2017-0242) was analyzed. Patients treated for early stages I–II NSCLC from 2014 to 2017 with definitive intent thoracic SBRT (BED_10_≥100) were included. Pretreatment baseline clinicopathologic characteristics expected to influence the development of acute lung toxicity were collected, including ECOG performance status, PFT parameters of FEV1, diffusing capacity for carbon monoxide (DLCO), tumor size, T stage, lobe of lung involved, and RTOG-defined central vs. peripheral locations. Treatment and dosimetric characteristics of dose, dose per fraction, prescription isodose line, and delivery schedule were also collected. Patients were scored as to whether corticosteroid prophylaxis was prescribed and utilized prior to each fraction. We recorded medication utilized, timing, and medication dose, and administration was confirmed through the electronic medical record (EMR).

Toxicity was graded on multiple domains including pulmonary domain using the Common Terminology Criteria for Adverse Events Version 4. Patients were scored as to whether they develop acute lung toxicity which was defined as grade 2 or higher pneumonitis or admission during or within 30 days of treatment completion. Hospitalization for acute exacerbation of COPD during or within 30 days after completion of treatment was also considered in the analysis. SBRT technique included simulation with helical 4-dimensional (4D) computed tomography (CT) scan utilizing respiratory imaging and synchronization *via* Real-Time Position Management System (Varian Oncology, Palo Alto, CA, USA). Patient immobilization and abdominal compression utilized the Civco Pro-Lok body system (CIVCO Medical Solutions, Orange City, IA, USA). An internal target volume (ITV) was delineated incorporating maximum inspiratory, maximum expiratory, as well as maximum intensity projection of the CT data. Uniform 5-mm planning target margin (PTV) was utilized around the ITV. Respiratory-gated treatment was employed if tumors demonstrated motion of greater than 1 cm. Static intensity-modulated radiation therapy and volumetric modulated arc therapy were employed for treatment delivery. All patients received daily volumetric image guidance with cone-beam CT, matching to the ITV.

Univariate analysis was performed with Fisher's exact test for categorical variables and two-tailed Student's t-test for continuous variables. Multivariate analysis was performed with Cox proportional hazards model to adjust for age, pretreatment DLCO, ECOG, tumor size, central versus peripheral location, and biological effective dose.

## Results

A total of 86 patients met the inclusion criteria for analysis. The median follow-up for the included cohort was 18.6 months.

Most patients received thoracic SBRT with 54–60 Gy in 3–8 fractions, with the majority (70%) receiving 5 fractions. Forty-five patients (52%) were treated with 4 mg dexamethasone premedication 30 min prior to each fraction of SBRT, and forty-one patients (48%) were treated without any dexamethasone premedication. Compliance with MD instructions for dexamethasone premedication was verified in the EMR.

**Table 1** summarizes baseline treatment, patient, and clinicoradiographic characteristics for the entire cohort and by pretreatment with dexamethasone.

**Table 1:** Basic patient clinical and treatment characteristics.

Patient and tumor characteristics	Entire cohort	Dexamethasone premedication	No premedication	*p*-value
**Patients [*n* (%)]**	86	45 (52%)	41 (48%)	
**Age (years)**				0.632^*^
Median	71	72	70	
Range	46–91	48–90	46–91	
**Median performance status (ECOG)**				0.321^*^
Median	1	1	1	
Range	0–3	0–3	0–3	
**RTOG tumor classification [*n* (%)]**				0.457^†^
Central	20 (23%)	12 (27%)	8 (20%)	
Peripheral	66 (77%)	33 (73%)	33 (80%)	
**Tumor size (cm)**				0.829^*^
Median	2.1	2.1	2.0	
Range	0.7–9.5	0.7–9.5	0.8–5.4	
**Tumor location**				1.000^†^
Upper lobes [*n* (%)]	44 (51%)	23 (51%)	21 (51%)	
Other [*n* (%)]	42 (49%)	22 (49%)	20 (49%)	
**Pretreatment pulmonary function**				
**DLCO (% Predicted)**				0.101^*^
Median	51	53	47	
Range	7–97	25–97	7–65	
**FEV1 (% predicted)**				0.461^*^
Median	56	58	50	
Range	24–138	30–117	24–138	
**Dose/fractionation**				
Average BED_10_ (Gy)	111.5	112.7	110.2	0.547^*^
5,400 cGy/3 fractions [*n* (%)]	5 (6%)	2 (4%)	3 (7%)	0.416^‡^
5,000 cGy/5 fractions [*n* (%)]	35 (41%)	22 (49%)	13 (32%)	
5,500 cGy/5 fractions [*n* (%)]	10 (12%)	4 (9%)	6 (15%)	
Other [*n* (%)]	36 (42%)	17 (38%)	19 (46%)	
**Median prescription isodose line (%)**	81	80	81	0.040^*^

ECOG, Eastern Cooperative Oncology Group; RTOG, Radiation Therapy Oncology Group; DLCO, diffusing capacity for carbon monoxide; FEV1, forced expiratory volume in 1 s; BED_10_, biologically effective dose with alpha/beta of 10; Gy, Gray.

^*^Student’s t-test.

^†^Fisher’s exact test.

^‡^Pearson’s χ^2^ test of association. Italics implies statistically significant since p is less then 0.05.

**Figure 1:**
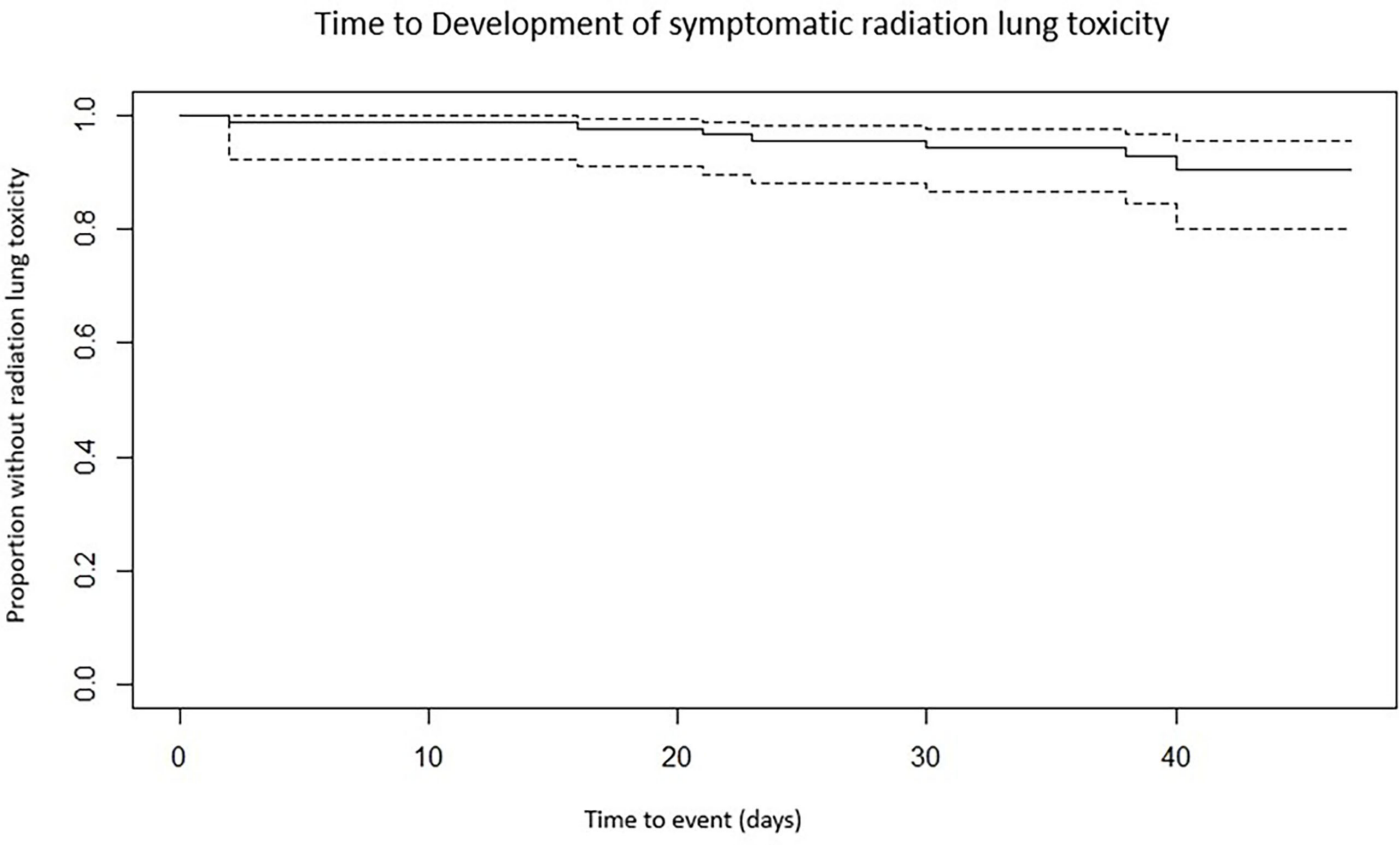
Kaplan–Meier estimate of time to development of acute lung toxicity for the entire cohort.

Overall acute lung toxicity was low in both groups, with seven patients (8.1%) experiencing grade 2 or greater acute pneumonitis or admitted to a hospital during or 30 days postcompletion of SBRT (see **Figure 1**).

Between the two groups, 5/45 (11%) patients developed grade 2 or higher lung toxicity including hospital admission in the dexamethasone premedication arm vs. 2/41 (5%) patients without premedication (*p* = 0.4370, Fisher’s exact test).

**Figure 2:**
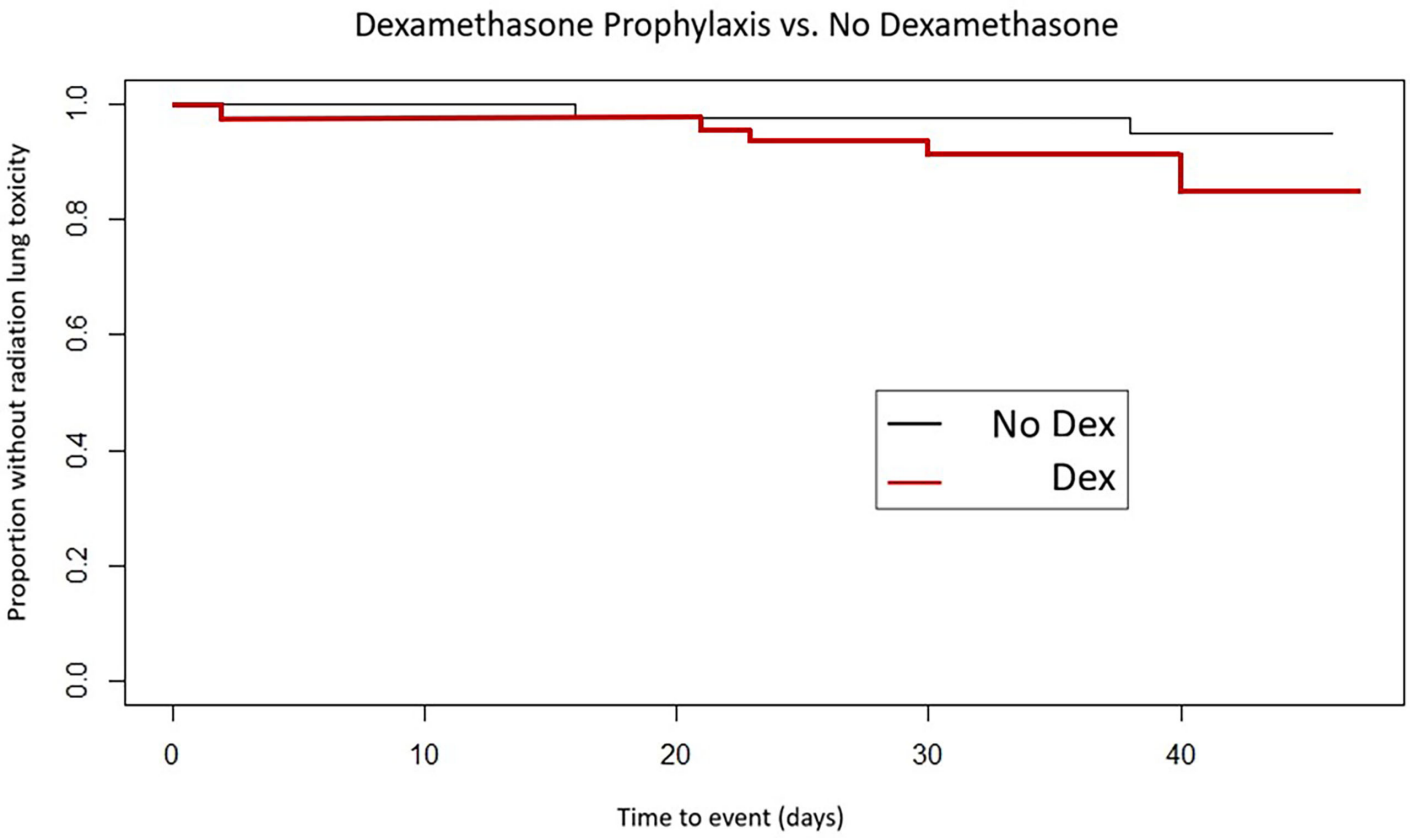
Time to development of acute lung toxicity by dexamethasone premedication vs. no premedication.

Freedom from acute SBRT lung toxicity was 91.1% in the dexamethasone-premedicated group vs. 97.6% in the group receiving SBRT without premedication (Log rank, *p* = 0.45) (see **Figure 2**).

On multivariate Cox proportional hazard modeling adjusting for age, ECOG performance status, tumor size, central vs. peripheral locations, pretreatment DLCO, dose per fraction, and biologically equivalent dose, there was no difference in freedom from acute lung toxicity with dexamethasone premedication (HR: 0.305; 95% CI: 0.033, 2.792; *p* = 0.293) (see **Table 2**).

**Table 2:** Freedom from development of acute lung toxicity controlling for other variables in multivariate model.

	HR (95% CI)	*p*-value
**Age (mean)**	1.028 (0.938–1.130)	0.558
**ECOG**	1.036 (0.324–3.317)	0.973
**0–1 vs. 2–3**		
**Tumor size [cm (mean)]**	0.996 (0.946–1.048)	0.884
**Pretreatment % predicted DLCO (mean)**	0.987 (0.952–1.021)	0.451
**RTOG definition**		
**Central vs. peripheral**	1.006 (0.678–1.491)	0.978
**Dexamethasone pretreatment**		
**Yes vs. No**	0.305 (0.033–2.792)	0.293
**BED_10_, Gy (mean)**	0.998 (0.995–1.000)	0.107

HR, hazard ratio; CI, confidence interval; ECOG, Eastern Cooperative Oncology Group; RTOG, Radiation Therapy Oncology Group; DLCO, diffusing capacity for carbon monoxide; BED_10_, biologically effective dose with alpha/beta of 10; Gy, Gray.

Significance determined using a Cox regression survival model.

## Discussion

Dexamethasone premedication did not appear to prevent against or mitigate acute lung toxicity or hospital admission during or in the subacute post-SBRT setting. To our knowledge, this represents the first report analyzing the influence of corticosteroid premedication in the development of acute lung injury in patients treated with thoracic SBRT.

Corticosteroid premedication has been utilized in large-dose per fraction stereotactic ablative approaches to the lung and abdominal sites since the advent of SBRT in the 1990s namely due to the bioplausibility of deterring acute inflammatory response in radiated tissue. The Karolinska Hospital experience first described stereotactic high-dose per fraction radiotherapy to the body and noted that acute high-grade toxicity could be mitigated with steroid premedication when treating tumors in the lung and liver (3, 4).

Clinical practice has been informed since then predominantly by its required use in the seminal RTOG 0236 phase II experience, but actual premedication use has been varied and largely based on institutional practice patterns. In addition, as lung SBRT cooperative group protocols have evolved, corticosteroid premedication has been increasingly left out or made optional at the discretion of the treating physician (11–20) (see **Table 3**).

**Table 3:** Incorporation of corticosteroid premedication evolution among clinical experience and cooperative group trials of thoracic SBRT.

Source	Year	Corticosteroid prophylaxis	Dose and Timing
Blomgren et al. (4)	1998	Mandatory	Dexamethasone or β-methasone
RTOG 0236 (5)	2004	Mandatory	Dexamethasone 4mg PO 15-60 minutes prior to each fraction
JCOG 0403 (11)	2004	N/A	N/A
RTOG 0618 (12)	2007	Recommended	Dexamethasone 4mg PO 15-60 minutes prior to each fraction
ROSEL Trial (13)	2008	N/A	N/A
RTOG 0813 (14)	2009	Not mandated or recommended, allowed at discretion of treating oncologist	N/A
RTOG 0915/NCCTG N0927 (15)	2009	Recommended	Dexamethasone 4mg PO 15-60 minutes prior to each fraction
RTOG 1021/ACOSOG Z4099 (16)	2012	Recommended	Dexamethasone 4mg PO 15-60 minutes prior to each fraction
SABR-COMET (17)	2012	N/A	N/A
NRG BR001/BR002 (18)	2014	Not mandated or recommended, allowed at discretion of treating oncologist	N/A
JoLT-Ca STABLE-MATES (19)	2015	Recommended	Dexamethasone 4mg PO 15-60 minutes prior to each fraction
NRG LU002 (20)	2017	N/A	N/A

Our study has several strengths. We were able to assess a relatively uniformly treated group of patients with similar disease and treatment characteristics in large part because our database spans an integrated healthcare system with 3 different treatment sites which employed different dexamethasone premedication based on prespecified site-specific SBRT protocol in terms of corticosteroid use and not based on patient selection. This allowed for a relatively uniform set of patients treated with similar SBRT technique, and steroid agent and dose were also uniform among the premedicated cohort. Nevertheless, the retrospective nature of the design does predispose to possible patient selection bias influencing the results. Multivariate analysis controlling for possible confounders, continued to show no added benefit to dexamethasone premedication. In addition, given the sample size of the study, it remains possible that small effect sizes from the influence of dexamethasone may not be adequately evaluated, and results should be confirmed in larger datasets.

Numerically, the steroid-premedicated arm had higher rates of hospitalization and acute lung toxicity. Although this was not statistically significant between groups, and may be largely influenced by selection bias, it is important to consider that dexamethasone use is not without inherent patient risks.

The side effect profile of corticosteroid use including effects on immunosuppression, hyperglycemia, electrolyte abnormalities, sleep disturbance, and gastric ulcer disease need to be carefully considered in light of our findings. The initial report of RTOG 0236, for example, did report one event of grade 4 hypocalcemia, largely felt to be related to steroid prophylaxis (5).

In addition, the emergence and increased integration of ablative radiotherapy with immune checkpoint inhibitors in NSCLC and oligometastatic progression to the lung, requires further careful consideration and judicious use of corticosteroid prophylaxis. Several reports have pointed to decreased efficacy of PDL1 blockade in patients receiving baseline corticosteroids, mechanistically related to a blunting of peripheral bursts of CD8-positive T cells needed in response to PDL1 blockade (21, 22). Adjuvant PDL1 blockage has shown significant survival benefits in more advanced lung cancer and is now being tested in earlier stages of NSCLC treated with SBRT (23, 24). Hence, our findings suggest significant information on this clinical scenario in patients planned to initiate PDL1 inhibition post-SBRT, showing that no significant safety signals were noted when employing high-dose per fraction lung SBRT without steroid prophylaxis. Further investigations tailoring radiation dose and fractionation schedule, for example, in the case of centrally located lesions, to minimize acute toxicity and optimize immunotherapy synergy may represent future avenues of investigation instead of relying on steroid premedication (25).

In this retrospective analysis, pretreatment steroid prophylaxis with dexamethasone confers a similar acute toxicity profile to treatment without steroid prophylaxis. The limitations of the study, including its retrospective nature, are that pre- and posttreatment pulmonary function tests and patient-reported symptomatic outcomes were not routinely and rigourously assessed. Further follow-up is needed, and the impact of steroid premedication on clinical (local control and survival) outcomes should be determined. These results should also be validated in independent datasets and prospectively with a patient-reported outcome instrument.

## Data Availability Statement

The raw data supporting the conclusions of this article will be made available by the authors, without undue reservation.

## Ethics Statement

The studies involving human participants were reviewed and approved by Geisinger Cancer Institute IRB. The patients/participants provided their written informed consent to participate in this study.

## Author Contributions

Conceptualization: FA and AM. Data curation: FA. Formal analysis: FA and PS. Investigation: FA, PS, and AM. Methodology: FA, PS, and AM. Resources: FA. Supervision: AM. Validation: FA, PS, and AM. Writing—original draft preparation: FA. Writing—review and editing: FA, PS, and AM. All authors contributed to the article and approved the submitted version.

## Conflict of Interest

The authors declare that the research was conducted in the absence of any commercial or financial relationships that could be construed as a potential conflict of interest.

## Publisher’s Note

All claims expressed in this article are solely those of the authors and do not necessarily represent those of their affiliated organizations, or those of the publisher, the editors and the reviewers. Any product that may be evaluated in this article, or claim that may be made by its manufacturer, is not guaranteed or endorsed by the publisher.
